# Hybrid Sponge-Like Scaffolds Based on Ulvan and Gelatin: Design, Characterization and Evaluation of Their Potential Use in Bone Tissue Engineering

**DOI:** 10.3390/ma13071763

**Published:** 2020-04-09

**Authors:** Leto-Aikaterini Tziveleka, Andreas Sapalidis, Stefanos Kikionis, Eleni Aggelidou, Efterpi Demiri, Aristeidis Kritis, Efstathia Ioannou, Vassilios Roussis

**Affiliations:** 1 Section of Pharmacognosy and Chemistry of Natural Products, Department of Pharmacy, National and Kapodistrian University of Athens, Panepistimiopolis Zografou, 15771 Athens, Greece; ltziveleka@pharm.uoa.gr (L.-A.T.); skikionis@pharm.uoa.gr (S.K.); eioannou@pharm.uoa.gr (E.I.); 2 Institute of Nanosciences and Nanotechnology, NCSR “Demokritos”, Aghia Paraskevi, 15310 Attiki, Greece; a.sapalidis@inn.demokritos.gr; 3 cGMP Regenerative Medicine Facility, Department of Physiology and Pharmacology, School of Medicine, Faculty of Health Sciences, Aristotle University of Thessaloniki, 54124 Thessaloniki, Greece; angelide@auth.gr (E.A.); kritis@auth.gr (A.K.); 4 Department of Plastic Surgery, School of Medicine, Faculty of Health Sciences, Papageorgiou Hospital, Aristotle University of Thessaloniki, 54124 Thessaloniki, Greece; demirie@auth.gr

**Keywords:** ulvan, gelatin, BDDE, 3D scaffolds, mesenchymal stem cells, bone tissue engineering

## Abstract

Ulvan, a bioactive natural sulfated polysaccharide, and gelatin, a collagen-derived biopolymer, have attracted interest for the preparation of biomaterials for different biomedical applications, due to their demonstrated compatibility for cell attachment and proliferation. Both ulvan and gelatin have exhibited osteoinductive potential, either alone or in combination with other materials. In the current work, a series of novel hybrid scaffolds based on crosslinked ulvan and gelatin was designed, prepared and characterized. Their mechanical performance, thermal stability, porosity, water-uptake and *in vitro* degradation ability were assessed, while their morphology was analyzed through scanning electron microscopy. The prepared hybrid ulvan/gelatin scaffolds were characterized by a highly porous and interconnected structure. Human adipose-derived mesenchymal stem cells (hADMSCs) were seeded in selected ulvan/gelatin hybrid scaffolds and their adhesion, survival, proliferation, and osteogenic differentiation efficiency was evaluated. Overall, it was found that the prepared hybrid sponge-like scaffolds could efficiently support mesenchymal stem cells’ adhesion and proliferation, suggesting that such scaffolds could have potential uses in bone tissue engineering.

## 1. Introduction

Biomaterials is a rapidly growing sector of materials, engineered especially to tackle therapeutic and diagnostic purposes. Natural polymers are considered an emerging technological platform for the development of such materials for biomedical applications. Their inherent biological properties, chemical tuning abilities, biocompatibility, biodegradability, sustainability, and eco-friendliness render them as promising alternative materials in this field in comparison to synthetic polymers [1,2]. The versatility of natural, and even more of modified polymers, is advantageous in the fields of targeted drug delivery, theranostics, and tissue engineering and they are therefore extensively utilized for the development of efficient, safe, and high quality products [3,4,5].

The marine ecosystem, due to its limitless biodiversity, has been proven an inexhaustible source of bioactive biomaterials [6,7,8,9]. Among them, marine polysaccharides are regarded as safe and non-immunogenic materials [10,11,12], while they are considered ideal for the development of novel systems for bioapplications, such as tissue engineering and drug delivery, due to the wide range of bioactivities they exhibit.

Ulvan is a water-soluble complex anionic sulfated polysaccharide, representing a major constituent of the cell walls of green seaweeds of the order Ulvales (Chlorophyta) [13,14]. Ulvan’s physical properties and pharmacological activity have been thoroughly investigated [15,16,17,18,19,20,21,22], with biological activities, such as antibacterial [23,24], antiviral [25,26], anticoagulant [27], antioxidant [28,29], and antihyperlipidemic [30], being frequently reported. Additionally, the intrinsic cytocompatibility of ulvan [31,32] highlights its suitability for the development of novel scaffolds. Nevertheless, the potential of ulvan as a biomedical polymer has not yet been exhaustively investigated. To date, a number of ulvan-based scaffolds [32,33,34,35,36,37], as well as polyelectrolyte complex materials [31,38,39], 2D crosslinked membranes [40] and polymer blended nanofibers [41,42] have been prepared.

Gelatin is a natural biopolymer obtained from the partial hydrolysis of collagens, the main protein constituting the connective tissues in vertebrate animals, which has been widely used as a biocompatible material due to its generally recognized safe status by the US Food and Drug Administration (FDA) [43]. Gelatin has been shown to form 3D ordered macroporous structures for applications in oral drug delivery and regenerative tissue engineering [44,45,46,47,48]. The characteristic triple-stranded helix structure of collagen is partially retained in gelatin. However, denaturation at room temperature—as well as partial degradation—weakens the mechanical properties of gelatin and limits its applications. For the reinforcement of gelatin materials, crosslinking and/or compositing with other materials have often been adopted [49,50,51,52,53,54,55,56,57]. In this direction, crosslinking using glutaraldehyde and genipin [58], transglutaminase [59], or ultraviolet light irradiation [60] has been achieved, offering 3D-shaped gelatin-based materials which are considered as promising candidates for cell culture.

Over the past years, efforts have focused on the development of biopolymer-based 3D scaffolds for a wide spectrum of biomedical applications. Both ulvan and gelatin have exhibited osteoinductive potential, either alone or in combination with other materials so as to prepare scaffolds for osseous tissue regeneration [31,34,36,38,43,56,61,62,63].

Herein, a series of 3D hybrid ulvan/gelatin (UG) scaffolds of variable weight ratios of ulvan to gelatin have been designed and prepared and their chemical and mechanical properties were characterized. Furthermore, a preliminary evaluation of cell adhesion, survival, proliferation, and osteogenic differentiation of human adipose-derived mesenchymal stem cells (hADMSCs) seeded on the ulvan/gelatin sponge-like scaffolds was undertaken in order to explore their potential use in bone tissue regeneration.

## 2. Materials and Methods

### 2.1. Materials

1,4-Butanediol diglycidyl ether (BDDE, ≥95%) was obtained from Sigma-Aldrich (Darmstadt, Germany). Alkaline processed gelatin (pH 3.8–7.6) was purchased from AppliChem (Darmstadt, Germany). Specimens of the green alga *Ulva rigida* were collected in the island of Evoia, Greece. The algal specimens were cleaned from epiphytes, rinsed with seawater and fresh water, and dried in continuous air flow. The dried alga was then cut in 1–5 mm pieces with a mill and stored at room temperature until used in paper bags in a dry, dark place. In order to obtain ulvan, 500 g of the air-dried alga were suspended in 10 L of dH_2_O and heated in an autoclave at 121 °C for 30 min. The hot aqueous solution was filtrated through cotton cloth and the filtrate was allowed to cool at room temperature. The extracted polysaccharide was then precipitated by the addition of 40 L ethanol (EtOH, 96% v/v). The resulting suspension was left at 4 °C overnight and the precipitate was filtered through cotton cloth, washed three times with EtOH, sonicated for 1 h in an ultrasonic bath, vacuum-filtered, and finally freeze-dried overnight to afford ulvan as an off-white powder. The chemical analyses of ulvan were performed as previously described [41].

### 2.2. Preparation of Chemically Cross-Linked Ulvan/Gelatin Hybrid Scaffolds

Gelatin and ulvan were dissolved in either acidic (1 mM HCl) or alkaline (40 mM NaOH) medium at 50 °C for 2 h to result in 3 wt % (w/v) solutions [40,64,65]. Gelatin solution was mixed with ulvan solution at weight ratios of 7:3, 5:5, and 3:7 and further stirred for another 4 h. Subsequently, BDDE (45 wt %) was added to the mixtures and the reaction was allowed to progress for 24 h at 50 °C. Afterwards, the solution was dialyzed against distilled water to remove any unreacted crosslinker, placed in an appropriate cylindrical mold with dimensions 5 (h) × 25 (d) mm at −20 °C overnight, and lyophilized at −110 °C for 24 h to allow for the formation of porous ulvan-based structures. Control scaffolds were obtained by crosslinking pure gelatin under the above-described conditions.

### 2.3. Scanning Electron Microscopy

The microstructure of the lyophilized UG scaffolds was investigated using a PhenomWorld desktop scanning electron microscope (SEM) (Phenom-World, Eindhoven, The Netherlands) with a tungsten filament (10 kV) and a charge reduction sample holder. The samples were inspected after sputter coating on a Mini Sputter Coater/Glow Discharge System SC7620 (Quorum Technologies Ltd, East Sussex, UK).

### 2.4. Determination of Porosity and Density

The porosity of the scaffolds was determined using the liquid displacement method [66]. Weighted (W) scaffolds were immersed in a known volume (V_1_) of EtOH in a graduated cylinder for 5 min and sonicated for 3 min. The total volume of the resulting mixtures was recorded as V_2_. Afterwards, the solvent-impregnated scaffolds were removed from the cylinder and the residual EtOH volume was recorded as V_3_.

The porosity (ε) of the scaffolds was calculated by the equation

ε (%) = [(V_1_ − V_3_)/(V_2_ − V_3_)] × 100 (1)

The density (d) of the scaffolds was calculated by the equation

d (g/mL) = W/(V_2_ − V_3_) (2)

where (V_1_ − V_3_) is the volume of EtOH retained within the scaffold and (V_2_ − V_3_) is the total volume of the scaffold. A minimum of three samples for each scaffold were analyzed.

### 2.5. FTIR Spectroscopy

A Fourier transform infrared (FTIR) spectrometer was used to evaluate the composition of UG scaffolds, as well as their degradation after incubation in phosphate buffered saline (PBS, pH 7.4) medium. All spectra were recorded on a Bruker Tensor 27 spectrometer (Bruker Optik GmbH, Ettlingen, Germany) at room temperature and averaged from 24 scans in the range of 500–4000 cm^−1^ with a resolution of 4 cm^−1^.

### 2.6. Thermogravimetric Analysis

Thermogravimetric analysis (TGA) was performed using a SETSYS Evolution 17 instrument (SETARAM INSTRUMENTATION, Caluire, France) under nitrogen flow. Ulvan, gelatin, and UG scaffolds of different compositions were heated from 25 to 800 °C at a heating rate of 10 °C min^−1^. Sample temperature, sample weight and heat flow were recorded continuously.

### 2.7. Determination of Water Uptake Ability

The water uptake ability of the sponge-like scaffolds was evaluated with pre-weighted lyophilized samples immersed in distilled water (1% w/v) and allowed to swell at RT. After each time point (30 sec, 1 h, 4 h, and 24 h), the bulk water was removed by placing the wet scaffold on a Petri dish for 1 min and subsequently the wet sample was weighted.

Water content in the swollen scaffolds was calculated by the equation

Water Uptake (%) = [(W_w_ − W_d_)/W_d_] × 100 (3)

where W_w_ is the weight of the wet scaffold and W_d_ is the initial weight of the dry scaffold. A minimum of three samples for each type/condition were analyzed.

### 2.8. In Vitro Degradation Study

Pieces of the scaffolds were immersed in PBS medium for 12, 24, and 96 h, at a concentration equal to 0.5% (w/v). After incubation at 37 °C, the scaffolds were washed twice with distilled water and freeze-dried. The observed changes in their weight were monitored. The chemical integrity of the various UG sponge-like scaffolds after 96 h of incubation was further evaluated with FTIR.

### 2.9. Measurement of Mechanical Compression

Samples, cylindrical in shape having dimensions of approximately 5 (h) × 25 (d) mm, were tested by mechanical compression experiments in a universal tensile testing machine (Z3, Thümler GmbH, Nürnberg, Germany) equipped with a Nordic Transducer load cell of 250 N. Compression testing was carried out as previously described [67] at a crosshead speed of 5 mm/min, until obtaining a maximum reduction in sample’s height of 80%. The compressive strength of the samples was determined as the stress at 50% height reduction. Compressive modulus is defined as the slope of the straight line obtained by linear regression of the stress–strain curve in the near elastic region of the material (between 0 and 1.0% strain). A minimum of five replicates were measured.

### 2.10. Statistical Analysis

The results were expressed as mean values ± SD. Statistical differences were estimated using analysis of variance (ANOVA) single factor test and were considered significant when *p* < 0.05.

### 2.11. Isolation, Culture, and Characterization of hADMSCs

Human adipose tissue (~70 mL) was harvested during a lipoaspiration procedure, under Papageorgiou Hospital Review Board approved protocols 263-7/12/2016 and patient informed consent. hADMSCs were isolated, expanded and characterized in the cGMP facility. Cells were isolated by enzymatic digestion using a mixed solution of collagenase/dispase (2 mg/mL each) and placed in a roller bottle for 4 h incubation at 37 °C, 5% CO_2_ in order to enhance tissue digestion. Cells were collected and expanded in MSC medium consisting of a-MEM, 15% FBS 2 mM glutamine, 0.1 mM *L*-ascorbic acid phosphate, 100 U/mL penicillin, and 100 mg/mL streptomycin. Isolated cells were characterized as mesenchymal stem cells using a Guava® easyCyte 8HT flow cytometer (Merck-Millipore, Darmstadt, Germany). Cells expressed CD90 and CD73 (positive markers), whereas CD45 was used as a negative marker. Unstained cells were used as a control to set the gates and analysis was performed as previously described [68].

### 2.12. Cell Seeding on Scaffolds

Scaffolds were sterilized in 70% EtOH for 30 min, washed with PBS, placed into 24-well plates and immersed in growth medium for 24 h before seeding to allow protein absorption on them. Cell seeding was carried out by pipetting 20 μL/scaffold of cell suspension (containing 5 × 10^5^ cells) and the scaffolds were incubated for 1 h at 37 °C, 5% CO_2_ to allow cell adhesion before the expansion medium was added (1.5 mL/scaffold). Cells were maintained in MSC for 7 days and medium was changed every 2 days for the duration of the experiment [69].

### 2.13. Ostegenic Differentiation of hADMSCs

Osteogenic differentiation was induced in 3D scaffold cultures in the presence of osteogenic medium, a-MEM supplemented with 15% FBS, 2 mM glutamine, 0.1 mM *L*-ascorbic acid phosphate, 100 U/mL penicillin, 100 mg/mL streptomycin, 10 nM dexamethasone (Sigma-Aldrich, St. Louis, MO, USA), 1.8 mM KH_2_PO_4_, and 5 mM glycerolphosphate (Sigma-Aldrich, St. Louis, MO, USA). After 7 days culture in MSC expansion medium the cell-seeded scaffolds were induced to differentiate into osteocytes by using osteogenic medium for 21 days (the medium was changed twice per week). The morphology of the UG scaffolds seeded with hADMSCs after 21 days culture in osteogenic medium was observed with SEM. Scaffolds were fixed with 3% v/v glutaraldehyde, rinsed, and then dehydrated in increasing concentrations (30–100% v/v) of EtOH in water. The samples were dried and observed without sputter coating.

### 2.14. Confocal Microscopy

Scaffolds seeded with hADMSCs were evaluated for cell viability and colonization after 7 days proliferation. The Viability/Cytotoxicity Assay Kit for Animal Live & Dead Cells was used (#3000, Biotium, CA, USA), according to the manufacturer’s instructions. Samples were visualized by a confocal upright fluorescence microscope (Nikon D-Eclipse 80i C1, Tokyo, Japan). For live/dead staining, hADMSCs/scaffold constructs were doubly stained with calcein AM and ethidium homodimer, staining living and dead cells, respectively. After adding both dyes, the constructs remained at RT in the dark for 30 min and were washed with PBS before visualization. The constructs were captured as z-stack images using the EZ–C1 FreeViewer (Ver3.20) software (Nikon, Tokyo, Japan). Quantification of fluorescence intensity was determined using ImageJ software (NIH, Bethesda, MD, USA). Corrected total cell fluorescence (CTCF) was calculated using the formula CTCF = Integrated Density − (Area of selected cell × Mean fluorescence of background readings) [70].

## 3. Results and Discussion

### 3.1. Crosslinking of Gelatin and Ulvan

In the present study, ulvan and gelatin crosslinking was performed by employing the bifunctional epoxide BDDE, which was selected due to its acceptability and applicability in biomedical, pharmaceutical and cosmetic applications. This reagent is already extensively used for hyaluronic acid crosslinking to produce commercially available stable fillers for dermatological purposes [71]. BDDE can react with different functional groups present within polysaccharidic or proteinaceous backbones, such as amino, hydroxyl, and carboxyl moieties [64,72,73,74,75]. It is well-documented that by varying the pH of the reaction media, different types of linkages between various reactive groups and the epoxy functionality may be formed.

**Figure 1:**
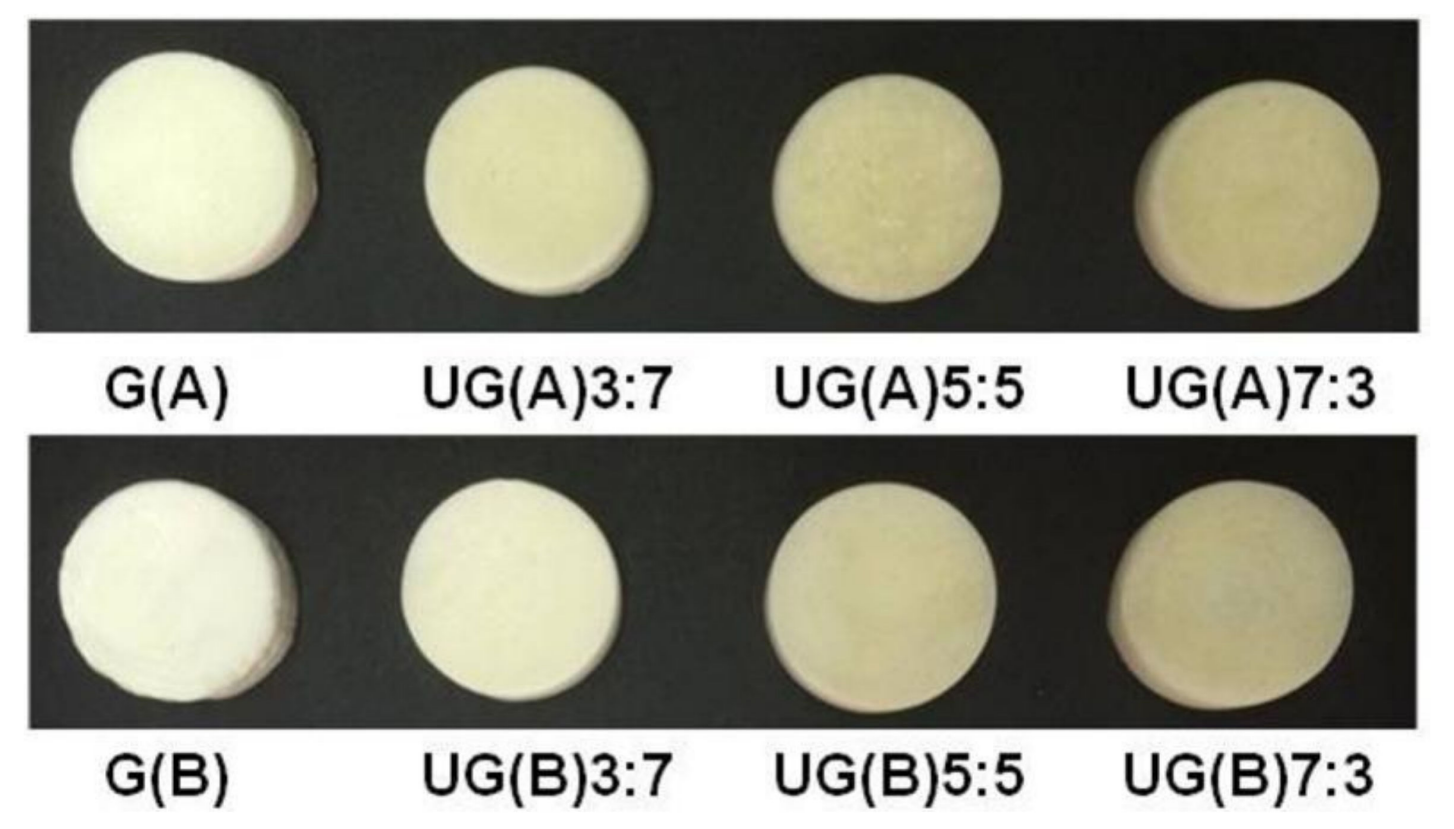
UG hybrid scaffolds of different composition prepared under acidic and basic conditions. The amount of ulvan and gelatin used are reported as w/w ratio.

**Scheme 1:**
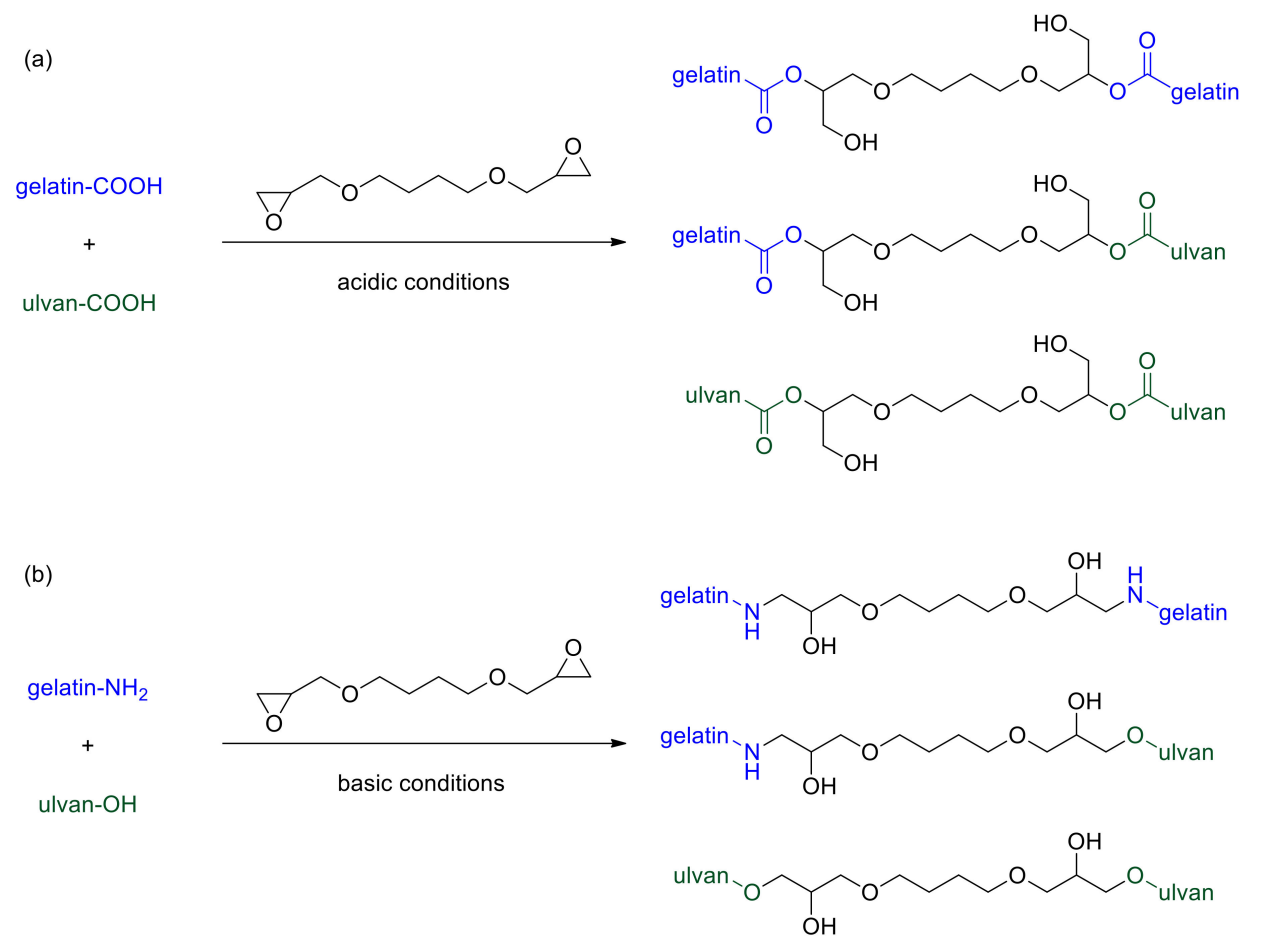
Schematic representation of the crosslinking of ulvan and gelatin with BDDE under (**a**) acidic and (**b**) basic conditions.

For the preparation of crosslinked gelatin and UG scaffolds, two different reaction conditions were employed. Crosslinking of either gelatin or ulvan/gelatin using BDDE was achieved under acidic, as well as basic conditions, yielding characteristic sponge-like scaffolds after lyophilization (Figure 1). Under acidic conditions, the carboxylic acid groups of both gelatin and ulvan—and to a much lesser degree the hydroxyl groups of ulvan—react with BDDE *via* an epoxide ring-opening mechanism, resulting mainly in the formation of ester bonds [64,73,74,75] (Scheme 1A). At basic pH values, BDDE is well documented to react primarily with the amine groups present in gelatin, and with the hydroxyl groups of ulvan [40,64,75,76] undergoing a ring-opening mechanism (Scheme 1B). BDDE concentration was kept constant (45 wt %) at all gelatin to ulvan weight ratios employed, in order to avoid the presence of unreacted crosslinker, which is prohibitive for biomedical applications.

Previous investigations showed that neat ulvan 3D porous structures present many limitations for tissue engineering applications [32] and ulvan concentrations higher than 4% (w/v) had to be employed in order to obtain scaffolds not readily soluble in PBS. In the current study, the addition of gelatin allowed for the formation of stable structures and 3D scaffolds even at final concentrations equal to 3% (w/v) for both polymers.

### 3.2. Characterization of Morphology and Porosity

**Figure 2:**
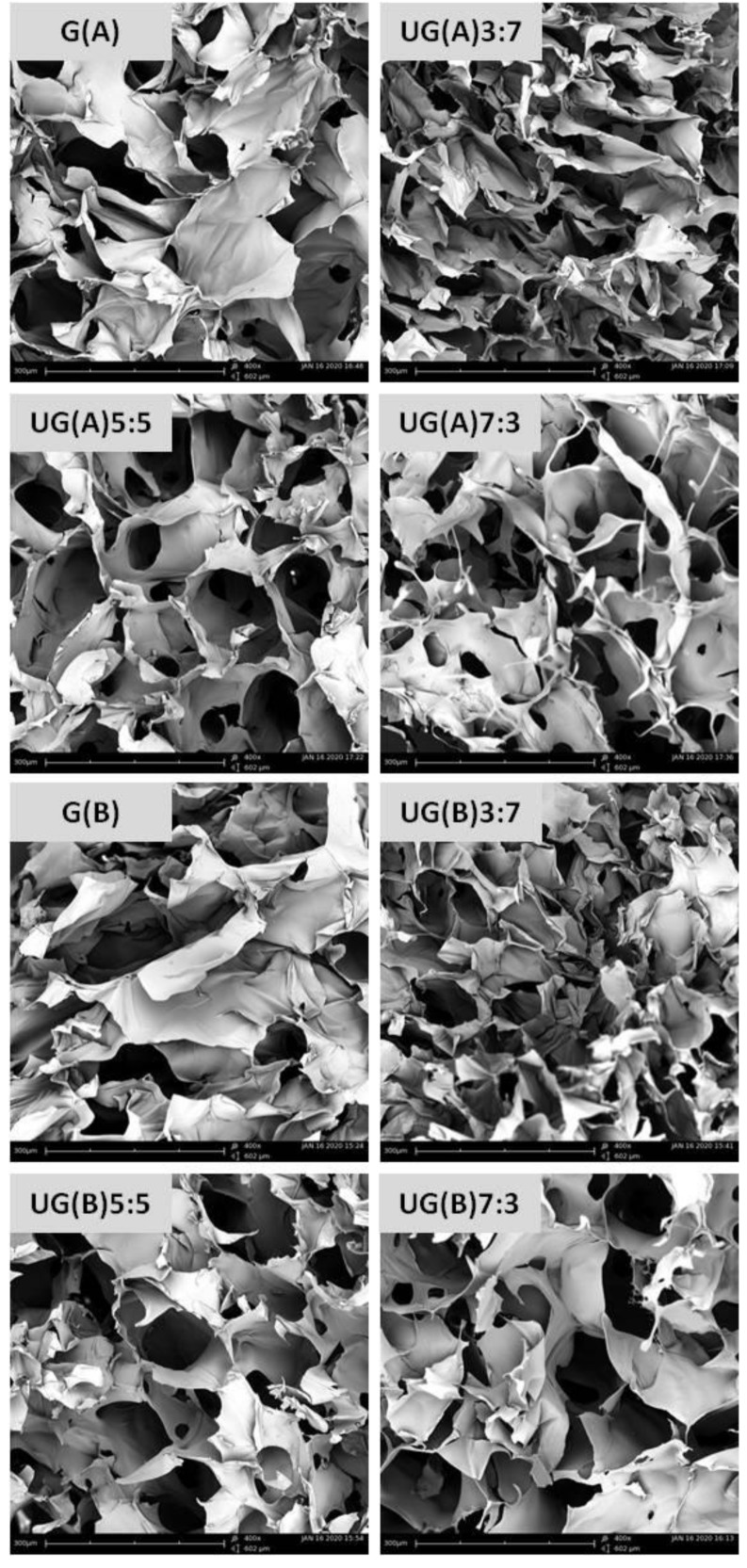
SEM images of UG hybrid scaffolds prepared under acidic or basic conditions and with varying ulvan to gelatin weight ratios.

The obtained sponge-like scaffolds were morphologically characterized by SEM. The morphology of the different hybrids was not significantly affected by the ulvan to gelatin ratios. All UG sponge-like scaffolds demonstrated an interconnected network structure between gelatin and ulvan, as depicted in a representative cross-section (Figure 2). Nevertheless, the pore homogeneity of the scaffolds differed, with the ones prepared under acidic conditions being less homogenous. The pore sizes ranged from 70 to 300 μm, with gelatin scaffolds exhibiting the largest pore sizes and UG3:7 scaffolds exhibiting the smallest. It is obvious that the addition of gelatin to ulvan affords structures with enhanced pore sizes, as compared to those previously reported for neat ulvan scaffolds (mean pore size 47 to 60 μm) [32].

It is well established that pore architecture is of paramount importance for the ability of scaffolds to provide a suitable support for cell adhesion, spreading, proliferation and finally tissue formation [77,78]. Pores that are too small and with limited interconnectivity restrain both cell colonization and proliferation. It has been reported that soft tissue biomaterials require interconnected pores of 100–300 μm to allow exchange of gases and nutrients and infiltration of cells. In the present study the optimal freezing temperature was determined to be −20 °C, at which open pores with an approximate size of 100 μm developed. The freezing temperature before lyophilization is known to have an impact on the characteristics of the resulting 3D scaffolds. In our case, the prepared samples were frozen at either −20 °C or −80 °C and the resulting structures after lyophilization exhibited open pore forms only when samples were frozen at −20 °C (data not shown). It has been previously suggested that, the number of nuclei of ice crystallization initially formed at higher freezing temperature is smaller than that at a lower freezing temperature, consequently leading to an increased final size of ice crystals. Larger ice crystals force polymer chains to expand to a greater extent, leading to increased pore size of the scaffolds [44,79].

The porosity of the prepared hybrid scaffolds was determined using the liquid displacement method. High porosities (>80%) were measured for all scaffolds (Table 1). Open-pore ratios above 85% are considered as indicative of well-interconnected porous structures, while high open-pore ratios are beneficial to cells and nutrients flow [43]. Furthermore, porosities above 70% are considered close to spongy bone (30–90% porosity) [80]. Hence, the obtained UG scaffolds with interconnected porous structure and high porosities could meet the requirements for bone tissue engineering. Moreover, the scaffolds prepared under both acidic and basic conditions were extremely light and their density remained almost unaffected regardless of the ulvan content (Table 1).

### 3.3. Infrared Spectroscopic Analysis

**Figure 3:**
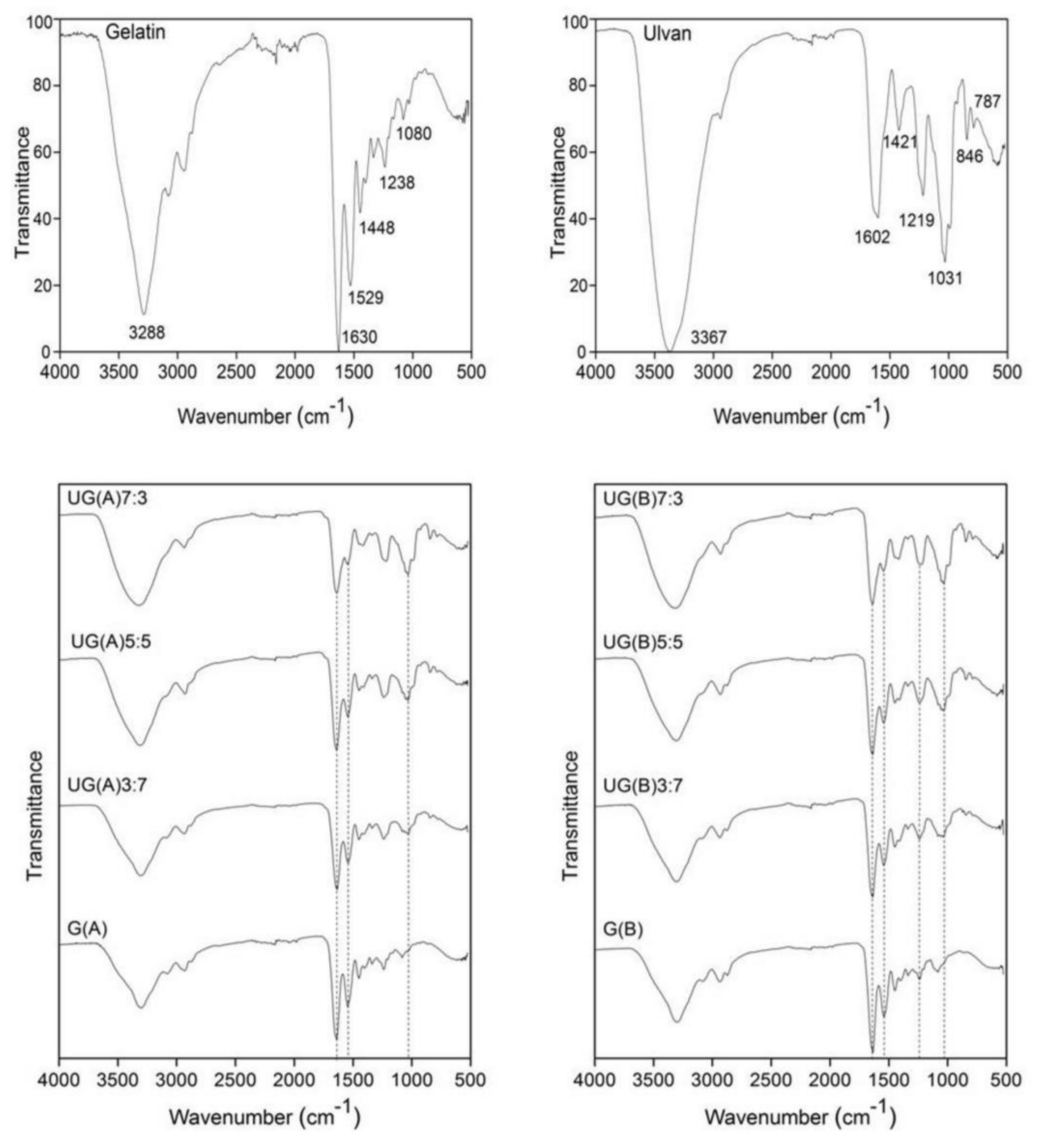
FTIR spectra of gelatin and UG scaffolds after crosslinking with BDDE. The FTIR spectra of gelatin and ulvan are also depicted for comparison reasons.

The successful incorporation of gelatin and ulvan in the prepared scaffolds was initially confirmed by ATR-FTIR spectroscopy (Figure 3). The FTIR spectrum of gelatin exhibits the characteristic intense absorption bands at 1630 and 1529 cm^−1^, attributed to amide I and amide II, as well as the observed absorption band at 1238 cm^−1^, assigned to amide III. The broad absorption band centred at 3288 cm^−1^ was assigned to –OH and –NH (amide A) stretching vibrations [81]. For ulvan, the most intense absorption bands were observed at 3367 and 1031 cm^−1^ attributed to the stretching vibration of OH groups and the strong stretching vibrations of C–OH groups and the ether glycosidic bridge (C–O–C), respectively. The carboxylate groups were evidenced by two absorption bands, an intense at 1602 cm^−1^ and a weaker one at 1421 cm^−1^, attributed to their asymmetric and symmetric stretching vibrations, respectively. In addition, the sulfate esters showed a strong absorption band at 1219 cm^−1^ and two more at 846 and 787 cm^−1^ [41,82,83,84].

After gelatin’s chemical crosslinking, both under acidic and basic conditions, the frequency of the signals attributed to amide A, amide I, amide II, and amide III remained nearly unchanged, indicative of the preservation of gelatin’s secondary structure. However, the integral absorbance (the area of the bands), especially of amide A, is smaller for crosslinked gelatin, suggesting that during the crosslinking reaction, gelatin loses bonded water [85].

The FTIR spectra of the hybrid UG scaffolds confirmed the presence of both gelatin and ulvan, by exhibiting their characteristic signals. As the ulvan content increases, stronger signals appear at approximately 1412, 1219, 1051, 1032, 845, and 785 cm^−1^ (characteristic signals of ulvan), verifying the larger amount of ulvan present in the scaffold. Moreover, when acidic conditions are employed for crosslinking, a detectable band at about 1734 cm^−1^, attributed to C=O stretching vibration is also observed [33]. Additionally, when crosslinking is undertaken under basic conditions, the expected signals at 1100 cm^−1^ related to the ether linkages formed cannot be traced due to their overlapping with the C–O–H and C–O–C absorption bands of ulvan [30,33].

### 3.4. Thermal Behavior

**Figure 4:**
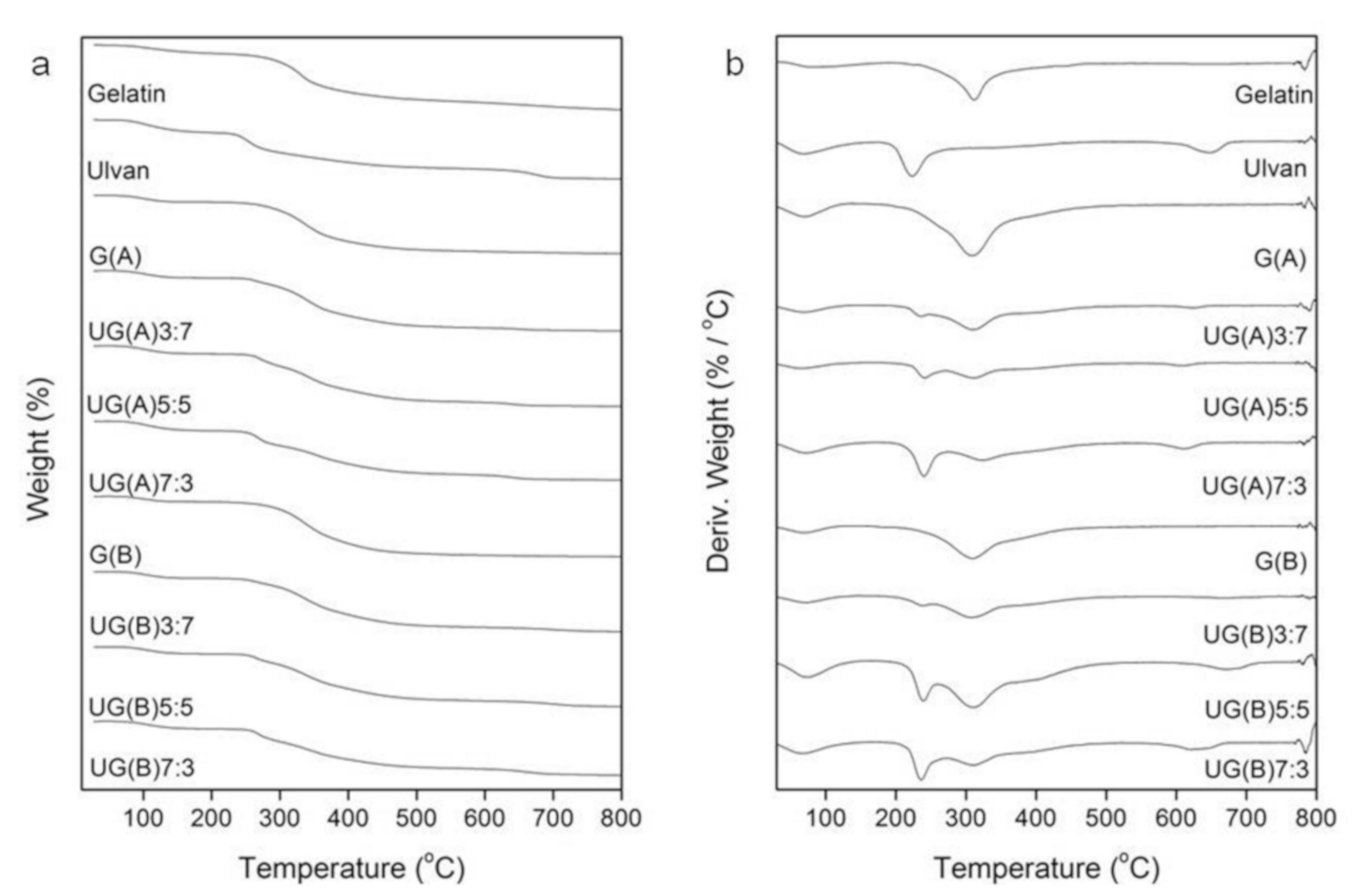
(**a**) TGA and (**b**) DTG thermogravimetric curves of gelatin, ulvan, and UG scaffolds.

The thermal properties of gelatin, ulvan, and the hybrid UG scaffolds were investigated by means of TGA which provides a quantitative measurement of mass change in materials associated with dehydration, decomposition, and oxidation of the sample as a function of time and temperature. The initial mass loss is generally attributed to the volatilization of the sample’s moisture, as hydrogen-bound water, related to desorption of bound water and dehydration reactions. Gelatin and ulvan characteristic thermogravimetric curves are presented in Figure 4. Both gelatin and ulvan present three degradation steps. Regarding gelatin, the first one, as already mentioned, corresponds to the elimination of absorbed and bound water. The second step is attributed to the decomposition of gelatin, as related to the main degradation and represents a complex process including protein chain breakage and peptide bond rupture. The final degradation step of gelatin can be attributed to the decomposition of more thermally stable structures produced after crosslinking reactions during heating [86,87]. On the other hand, the second decomposition step of ulvan occurs at an inflection point of 224 °C and indicates the decomposition of ulvan by the dehydration of the sugars, breaking up of C–H bonds and breaking of the C–O–C glycoside bonds in the main polysaccharide chain. Therefore, the thermal stability of ulvan is ascertained until about 200 °C. The third and final degradation step can be attributed to the inorganic material present in ulvan as counterions associated with sulfate groups and uronic acid [16]. Moisture associated with gelatin and ulvan approximates 11% and 17%, respectively.

The consistency of the hybrid UG scaffolds (Figure 4) was also confirmed by the fact that the patterns of the TG curves of UG scaffolds depend on the concentration of each component during the degradation process. The temperature of the maximum speed of the process (*T*_max_) was determined from the maximum on the DTG curve. The decomposition curves of the hybrid UG scaffolds demonstrate that while the maximum rate of the degradation process of the gelatin component practically remains the same (~315 °C), that of the ulvan component increases, relatively to the ulvan content (from 224 to 241 °C). Additionally, the intensity of the respective DTG curves increase parallel to the gelatin to ulvan weight ratio employed. The fact that two DTG curves are observed points to the existence of a gelatin and an ulvan–dominating scaffold with no uniform composition.

### 3.5. Water Uptake Ability

Scaffolds with high water content are considered to mimic fundamental characteristics of the extracellular matrix (ECM) and hence have the potential to direct cell migration, growth and organization during tissue regeneration and wound healing. Such scaffolds are extremely compatible to certain native tissues, such as cartilage, cornea, liver, nerve, and bone [80,88].

**Figure 5:**
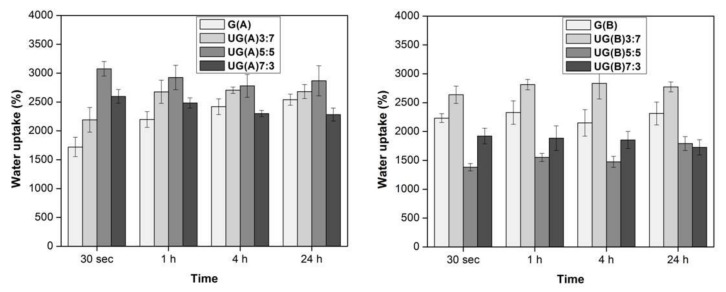
Water uptake ability (%) of UG scaffolds as a function of time.

The water uptake ability of the ulvan/gelatin scaffold is depicted in Figure 5. The scaffold prepared under both conditions absorbed water within 30 s. Specifically, the hybrid UG(A) scaffold prepared under acidic conditions became saturated within 30 s. Increase in the ulvan content up to a 1:1 U/G w/w ratio [UG(A)5:5] increased in parallel the water uptake, resulting to the highest water uptake observed (3000%). Further ulvan content increase led to a reduction of the water uptake. Instead, the G(A) scaffold needed at least 1 h in order to become saturated.

When basic conditions were employed for the scaffold preparation, all types of scaffolds became saturated within 30 s. The water uptake of the hybrid UG(B) scaffolds was once again dependent on the ulvan content. Once ulvan content exceeded the ratio of 1:1 U/G w/w ratio [UG(B)5:5], the uptake was decreased. The highest water uptake was observed for the UG(B)3:7 scaffold (2800%). The G(B) scaffold exhibited a swelling profile almost comparable to that of UG(B)3:7. Overall, limited addition of ulvan improves the water uptake efficiency of the hybrid scaffolds, as compared to neat crosslinked ulvan 3D structures (2000%) [32].

During the crosslinking process no hydrogen bonding groups (N–H, OH, and COOH) are eliminated, and therefore, no change in water bonding is expected. Consequently, the observed differences in water uptake pattern, since they cannot be attributed to increasing porosity of the sponge-like scaffolds by adding ulvan, might be due to changes in the hydrophilicity of the contact surface area. Most probably, by increasing ulvan content, a decrease in the hydrophilicity is accomplished owing to the amphiphilic character of ulvan [89]. This does not occur at the same degree in both UG(A) and UG(B) scaffolds, pointing to variations in the ratios of the different conjugates (ulvan–ulvan, ulvan–gelatin, and gelatin–gelatin) composing the scaffolds (Scheme 1).

### 3.6. In Vitro Biodegradability

**Figure 6:**
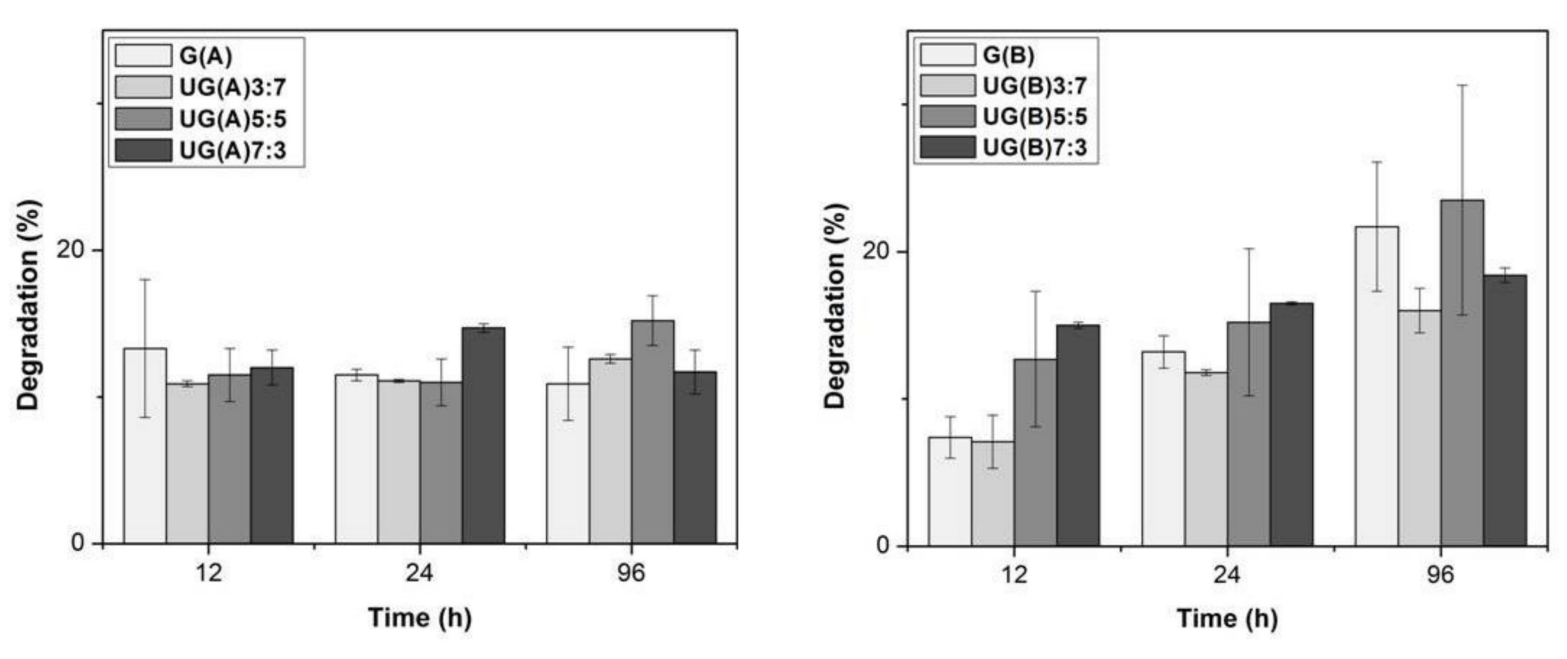
Degradation (%) of UG scaffolds as a function of incubation time at 37 °C.

The degradation behavior as a function of U to G ratio and the crosslinking conditions employed was investigated. The percentage of weight loss of crosslinked scaffolds as a function of degradation time is presented in Figure 6. In the case of the crosslinked under acidic conditions scaffolds, the weight loss remained constant up to 96 h of incubation at 37 °C and equal to approximately 15%. The ratio of U to G in the scaffolds did not seem to affect the weight loss rate. Based on these results, the limited rate of weight loss of ulvan/gelatin scaffolds was considered to depend mainly upon the crosslinking conditions rather than the mixing ratios of the scaffolds’ constituents.

Under basic conditions, the weight loss increased slightly in parallel to the incubation time at 37 °C, increasing from approximately 10 to 20% after a 12 to 96 h incubation period, respectively. In this case, the ratio of the different constituents of the scaffolds affected the weight loss rate at short incubation time. Specifically, after a 12 h incubation, the higher the ulvan content, the higher the degradation observed. At longer incubation periods no such differences were observed. The above-mentioned results indicate that crosslinked under basic conditions gelatin is initially more resistant to degradation in PBS solution. Overall, irrespective of ulvan/gelatin mixing ratios, the crosslinked scaffolds showed limited degradation during the period of 96 h.

**Figure 7:**
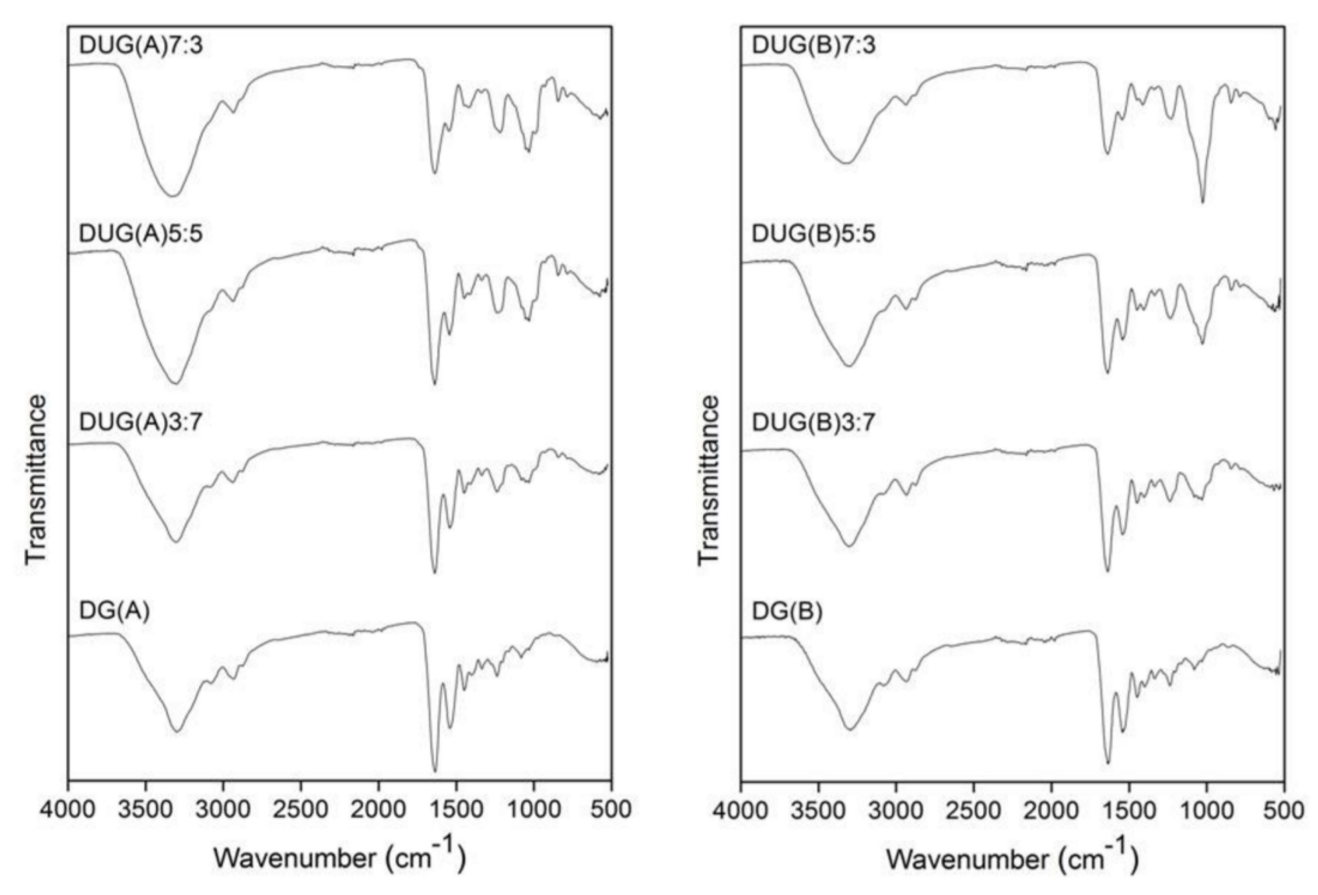
FTIR spectra of the recovered UG scaffolds after incubation in PBS at 37 °C for 96 h.

In order to examine whether the composition of the scaffolds was retained, the scaffolds obtained after a 96 h incubation period were washed with deionized water, lyophilized and their FTIR spectra were recorded (Figure 7). No preference in the degradation of the constituents of the UG scaffolds obtained under acidic conditions was observed. For the UG scaffolds obtained under basic conditions, differences were recorded concerning the relative intensity of the signal resonating at 1031 cm^−1^, characteristic of ulvan (see above). The signal intensity increased after the treatment, suggesting the preferential removal of gelatin.

Most probably, mass loss originates from the dissolving/leaching out of the soluble fractions, consequently resulting to a decreased swelling ratio. As such, lost mass as a result of degradation creates more space that can accommodate the growth of new cells and the deposition of ECM [56].

### 3.7. Mechanical Properties

It is well established that a porous structure weakens the mechanical strength of a scaffold [63]. The compression testing of the UG(A) and UG(B) scaffolds revealed typical sponge-like behavior (see Supplementary Materials Figure S1). As seen in Table 2, the samples prepared under acidic conditions exhibited an almost identical Young’s modulus under compression in the order of 0.1 MPa, while their compressive strength at 50% height reduction, was in the range of 0.043 MPa for UG(A)7:3 to 0.074 MPa for UG(A)3:7. In the case of samples crosslinked under basic conditions, differences in their mechanical behavior were apparent. UG(B)3:7 exhibited the best performance in both compression Young’s modulus and strength (0.19 and 0.09 MPa, respectively). Alves et al. [32] reported on the preparation of 3D crosslinked ulvan scaffolds and demonstrated that both crosslinker and ulvan concentrations influenced the properties of the produced structures. Overall, low polysaccharide concentrations (4% w/v) resulted in scaffolds readily soluble in aqueous media. In our case, we observed that the addition of gelatin to ulvan solutions resulted to an increase of the mechanical properties of the hybrid scaffolds, even when prepared from low concentration solutions. The trend in the mechanical properties in the samples prepared under basic conditions is the UG(B)3:7 > G(B) > UG(B)5:5 > UG(B)7:3 from stiffest to softest. Our results show that addition of limited amount of ulvan to alkaline solutions of gelatin resulted in scaffolds with improved mechanical properties. However, further increase of ulvan content led to scaffolds of poor quality. Soft tissue biomaterials need to be pliable to match the contours of the tissue defect, while offering adequate mechanical support to prevent the collapse of the defect. Stiffness values of soft tissue vary greatly between different tissue types, disease state, and testing modality; however, most mammalian soft tissues have elastic moduli between 0.1 and 100 kPa [90].

### 3.8. Evaluation of Cell Viability, Proliferation, and Morphology of hADMSCs Seeded on the Scaffolds

**Figure 8:**
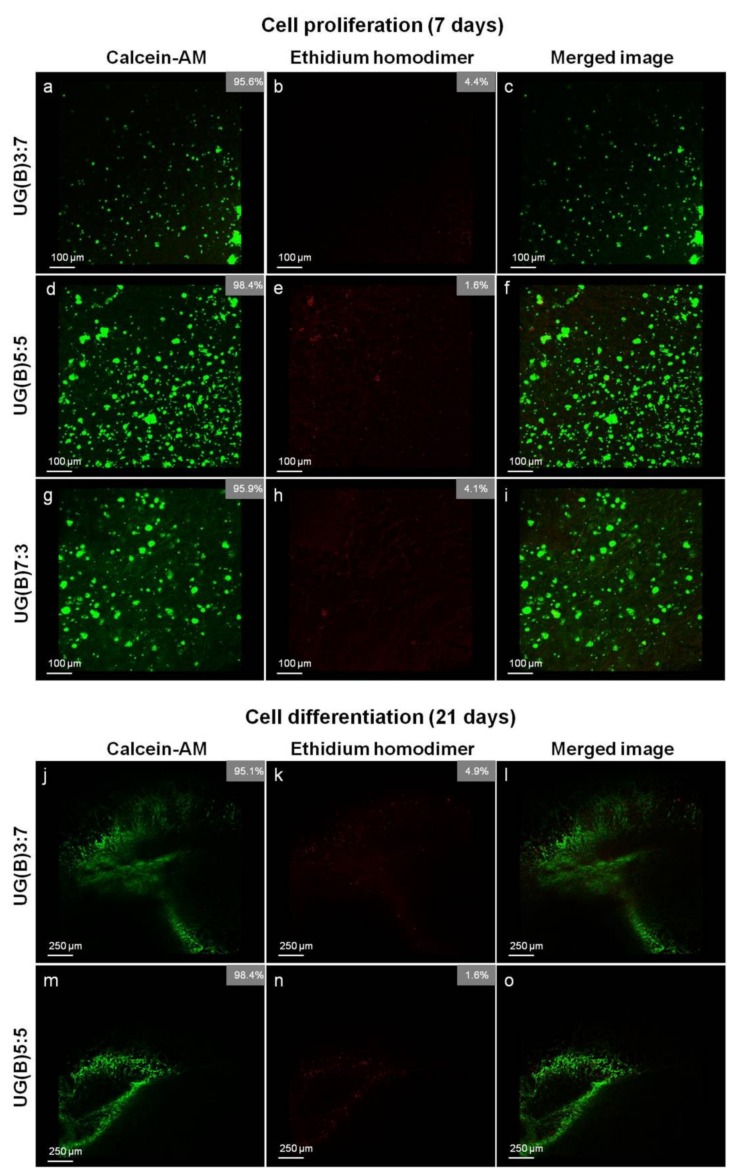
Confocal images of UG(B) scaffolds seeded with hADMSCs. Live/dead staining with calcein AM/ethidium homodimer showing cell viability of hADMSCs (**a**–**i**) after 7 days culture and (**j**–**o**) after 21 days culture in osteogenic medium. The relative cell viability or mortality (%) are denoted in the upper right part of the images.

Cell adhesion and viability was evaluated by confocal microscopy. Most cells seeded on selected UG hybrid scaffolds remained viable during the 7 days of MSC expansion, appearing as sparse round clusters, probably originating from single-cell-formed colonies (Figure 8a–i). In contrast, very few dead cells were observed as red rounded bodies. The cells cultured on UG(B)5:5 scaffold showed enhanced viability and spreading, as confirmed by their uniform distribution on the surface of the scaffold. Samples UG(B)7:3 and especially UG(B)3:7 induced the attachment of a lower number of cells. After 21 days of cultivation in osteogenic medium, the majority of the cells remained viable and appeared to evenly colonize a wide area of the UG(B)3:7 and UG(B)5:5 scaffolds (Figure 8j–o), with cell viability at 95.1% and 98.4%, respectively. In the case of the UG(B)7:3 scaffold, cell viability could not be determined, since after 21 days it was decomposed to a significant degree. As it can be observed, UG(B) scaffolds support both cell viability and growth.

### 3.9. Evaluation of Osteogenic Differentiation of hADMSCs Seeded on the Scaffolds

**Figure 9:**
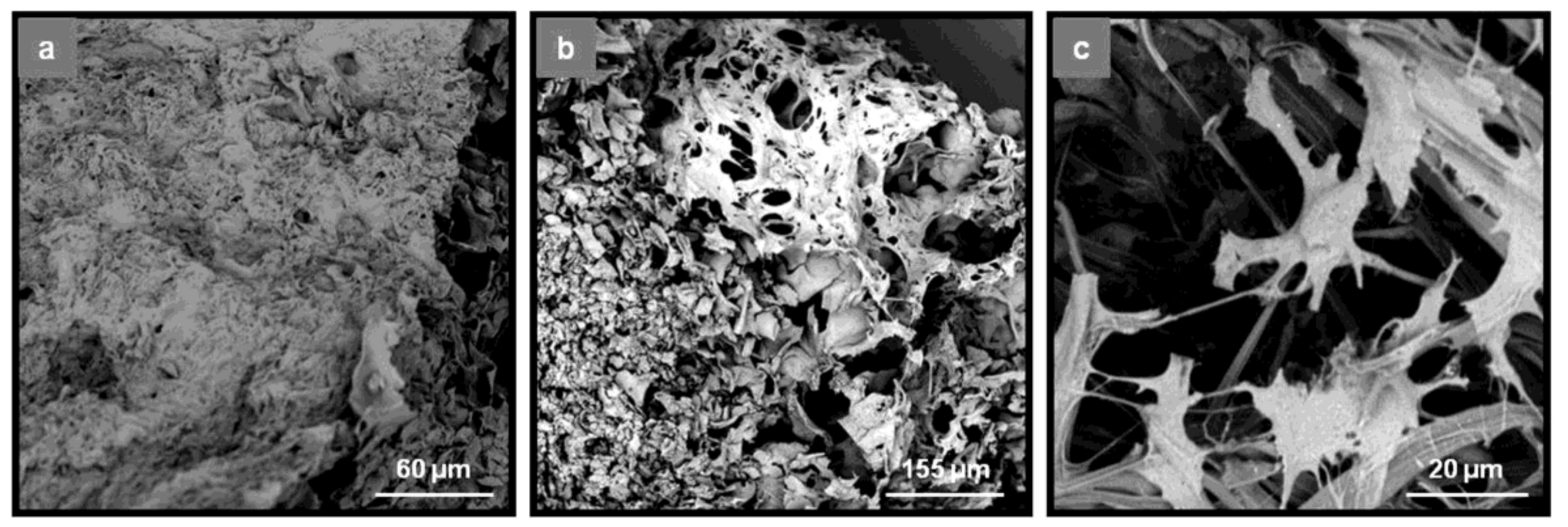
SEM images of UG(B) scaffolds seeded with hADMSCs, depicting the adhesion and spreading of hADMSCs inside the porous structure of the scaffolds after 21 days culture in osteogenic differentiation medium. (**a**) UG(B)3:7; (**b**) UG(B)5:5; and (**c**) magnification of UG(B)5:5 showing the changing morphology of hADMSCs towards osteogenic differentiation.

Scanning electron microscopy was used to assess the morphology and the degree of osteogenic differentiation of hADMSCs seeded on the selected ulvan/gelatin scaffolds, after 21 days of exposure to osteoinductive growth medium (Figure 9a,b). hADMSCs remained viable after 21 days in culture and underwent dramatic morphological changes. When grown in isolated patches, they displayed complex, radiant, spike-like cytoplasmic trabeculae, forming bridges with neighboring cells, characteristic of the marked phenotype for osteogenic differentiation (Figure 9c). When small in number, this phenomenon resulted in a cell layer with empty spaces. When in large numbers, the empty spaces were filled and the cells appeared to colonize the scaffold in wide areas, forming an extended layer of extracellular matrix.

## 4. Conclusions

Within the framework of our research interests in the preparation of ulvan-based biomaterials with potential biomedical applications, hybrid ulvan/gelatin sponge-like scaffolds have been prepared and their potential application as bone tissue engineering materials was evaluated. The different hybrid UG scaffolds showed substantial variation in their stability, enhanced water uptake ability, and high porosity, but poor mechanical properties. Although SEM analysis revealed small variation in the morphology of the prepared hybrid UG scaffolds, disclosing well-interconnected porous structures with pores of comparable size, confocal images displayed an altered expression on cell attachment and viability of hADMSCs. It seems that the differential ulvan to gelatin ratio affects cell adhesion and proliferation, with scaffolds prepared on a 1:1 ratio exhibiting improved cell viability. SEM analysis of the seeded with hADMSCs UG scaffolds after 21 days of exposure to osteoinductive growth medium indicated osteogenic differentiation, as deduced by the morphological changes of the cells that displayed irregular shape, with small cell bodies and spikes, forming bridges with neighboring cells. Optimization of the ulvan/gelatin 3D scaffolds could improve their stability and mechanical properties, while retaining or further enhancing cell attachment, viability, proliferation, and differentiation, rendering them a promising construct for bone tissue engineering.

## Figures and Tables

**Table 1:** Porosity and density of UG scaffolds

Sample	Porosity (%)	Density (g/mL)
G(A)	87.5 ± 1.6	0.030 ± 0.002
UG(A)3:7	85.2 ± 2.5	0.030 ± 0.002
UG(A)5:5	95.3 ± 0.7	0.031 ± 0.001
UG(A)7:3	91.5 ± 4.6	0.034 ± 0.004
G(B)	84.6 ± 4.5	0.031 ± 0.006
UG(B)3:7	82.3 ± 4.2	0.031 ± 0.005
UG(B)5:5	93.7 ± 3.7	0.031 ± 0.003
UG(B)7:3	92.1 ± 2.9	0.033 ± 0.001

**Table 2:** Young’s modulus and compressive strength at 50% compression for UG scaffolds

Sample	Young’s Modulus (Compression, MPa)	Compressive Strength at 50% Compression (MPa)
G(A)	0.10 ± 0.018	0.068 ± 0.010
UG(A)3:7	0.08 ± 0.01	0.074 ± 0.009
UG(A)5:5	0.09 ± 0.006	0.059 ± 0.008
UG(A)7:3	0.08 ± 0.015	0.043 ± 0.002
G(B)	0.06 ± 0.005	0.050 ± 0.010
UG(B)3:7	0.19 ± 0.005	0.090 ± 0.010
UG(B)5:5	0.04 ± 0.014	0.030 ± 0.005
UG(B)7:3	0.02 ± 0.0003	0.020 ± 0.003

## Supplementary Materials

The following are available online at https://www.mdpi.com/1996-1944/13/7/1763/s1, Figure S1: stress–strain curves of the UG scaffolds.
