# Supplementary material for: Hybrid Sponge-Like Scaffolds Based on Ulvan and Gelatin: Design, Characterization and Evaluation of Their Potential Use in Bone Tissue Engineering
Source: Materials (Basel). 2020 Apr 9;13(7):1763. doi: 10.3390/ma13071763 (PMC7178717; doi:10.3390/ma13071763)
Supplement: Supplementary file 1 [file materials-13-01763-s001.pdf]

materials

*Supplementary Materials*

# Hybrid Sponge-Like Scaffolds Based on Ulvan and Gelatin: Design, Characterization and Evaluation of Their Potential Use in Bone Tissue Engineering

**Leto-Aikaterini Tziveleka [1], Andreas Sapalidis [2], Stefanos Kikionis [1], Eleni Aggelidou [3], Efterpi Demiri [4], Aristeidis Kritis [3], Efstathia Ioannou [1] and Vassilios Roussis [1,*]**

[1] Section of Pharmacognosy and Chemistry of Natural Products, Department of Pharmacy, National and Kapodistrian University of Athens, Panepistimiopolis Zografou, Athens 15771, Greece; ltziveleka@pharm.uoa.gr (L.-A.T.); skikionis@pharm.uoa.gr (S.K.); eioannou@pharm.uoa.gr (E.I.)
[2] Institute of Nanosciences and Nanotechnology, NCSR "Demokritos", Aghia Paraskevi, 15310 Attiki, Greece; a.sapalidis@inn.demokritos.gr
[3] cGMP Regenerative Medicine Facility, Department of Physiology and Pharmacology, School of Medicine, Faculty of Health Sciences, Aristotle University of Thessaloniki, Thessaloniki 54124, Greece; angelide@auth.gr (E.A.); kritis@auth.gr (A.K.)
[4] Department of Plastic Surgery, School of Medicine, Faculty of Health Sciences, Papageorgiou Hospital, Aristotle University of Thessaloniki, Thessaloniki 54124, Greece; demirie@auth.gr
* Correspondence: roussis@pharm.uoa.gr; Tel.: +30-210-727-4592

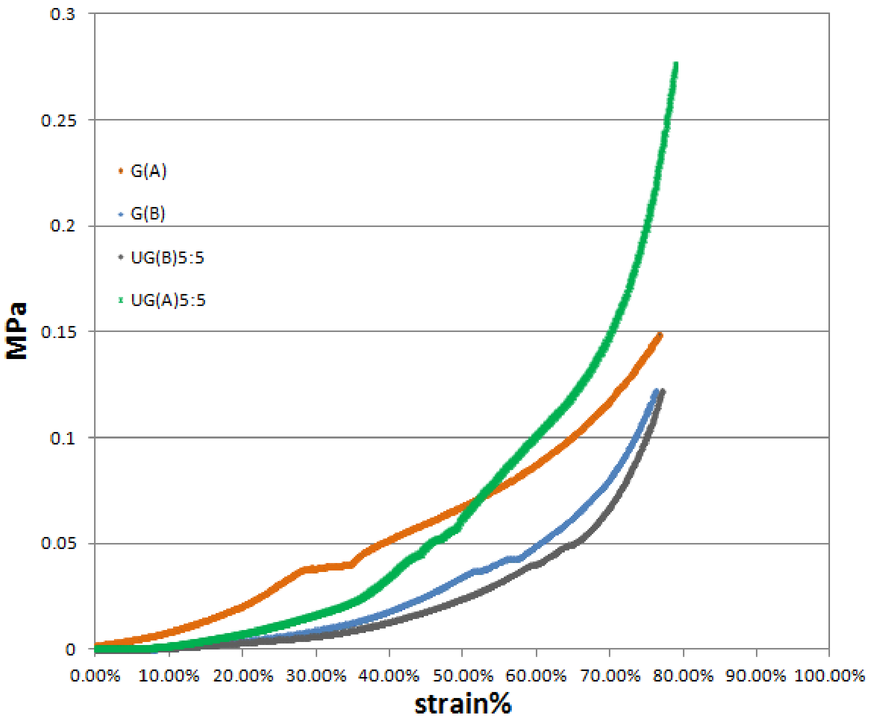


**Figure S1.** Representative stress-strain curves of UG scaffolds.

© 2020 by the authors. Submitted for possible open access publication under the terms and conditions of the Creative Commons Attribution (CC BY) license (http://creativecommons.org/licenses/by/4.0/).
